# Supplementary figures and images for: A rad50 germline mutation induces tumorigenesis and ataxia-telangiectasia phenotype in a transparent medaka model
Source: PLoS One. 2023 Apr 25;18(4):e0282277. doi: 10.1371/journal.pone.0282277 (PMC10129005; doi:10.1371/journal.pone.0282277)

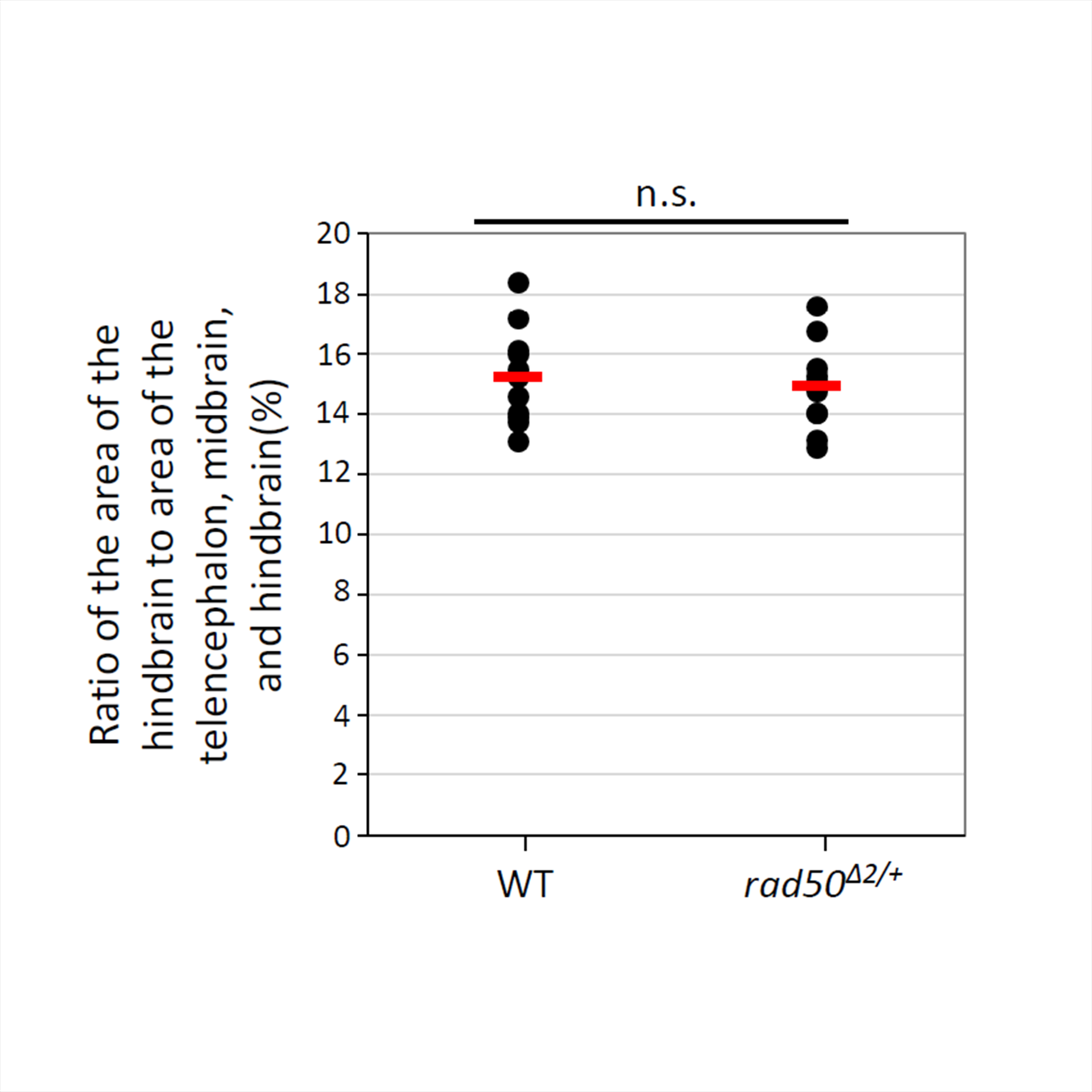

Supplement: S1 Fig — A ratio of the area of the hindbrain to area of the telencephalon, midbrain, and hindbrain in wildtype (WT) and rad50Δ2/+ medaka. The areas were calculated using images. The black dots and short horizontal red lines represent the data obtained from one individual and the median, respectively. n.s. indicates the absence of a statistically significant difference (Mann–Whitney U test). (TIF) [file pone.0282277.s001.tif]
