# Supplementary material for: A rad50 germline mutation induces tumorigenesis and ataxia-telangiectasia phenotype in a transparent medaka model
Source: PLoS One. 2023 Apr 25;18(4):e0282277. doi: 10.1371/journal.pone.0282277 (PMC10129005; doi:10.1371/journal.pone.0282277)
Supplement: S1 Table — (DOCX) [file pone.0282277.s002.docx]

S1 Table. PCR and sequencing primers.

| Primer name | Sequences* (5ʹ-3ʹ) |
| --- | --- |
| Primer 1 | TGATTGATCCCTTTTAGAAAAGACCTTGTG |
| Primer 2 | GCTCCTCTTTGTCCAGCTGGAA |
| Primer 3 | GATTGATCCCTTTTAGAAAAGACCTTGTG |
| Primer 4 | TGAGGAAATAAGACTATCAGTGAGACAAC |
| Medμ 45U | **FAM-**GTTTGGTGATGGGAGAAGAATAGT |
| Medμ 45L | TGACAAAAGGTGAGCAATAG |
| Medμ 52U | **VIC**-GATTGGACCTGTGACTCTAA |
| Medμ 52L | ATCTCTTGAAGGGTTTATACTG |
| Medμ 60U | **NED**-CCAGGAGAGGAATAATAAAC |
| Medμ 60L | TGGGAAAAGTGCTGTATAA |
| Medμ 58U | **PET**-TCACCAGTAGAAAACTTTACAG |
| Medμ 58L | AATGTCATAAAAAAGGGACTAA |
| *FAM, VIC, NED, and PET are fluorescent dyes. | |
