# Supplementary material for: A rad50 germline mutation induces tumorigenesis and ataxia-telangiectasia phenotype in a transparent medaka model
Source: PLoS One. 2023 Apr 25;18(4):e0282277. doi: 10.1371/journal.pone.0282277 (PMC10129005; doi:10.1371/journal.pone.0282277)
Supplement: S1 Information — DeepLabCut (version 2.0) was used to create an analytical model of medaka swimming and to extract the coordinates of the head and body positions. First, 19 frames were automatically extracted using the “kmeans” algorithm from the video of one wildtype medaka. In these frames, the head position was labeled between the left and right eyes. The body position was labeled between the left and right pectoral fins. The size of each marker was 5 μm. Nineteen frames were used as the training dataset in DeepLabCut. Next, DeepLabCut was trained 50,000 times to recognize the position of the head and body of the medaka in the movie based on the training dataset, and an analysis model of medaka swimming was generated. The analytical model of medaka swimming was used to analyze all movies of wildtype and rad50Δ2/+ medaka, and the head and body coordinates were extracted. The root mean square values were calculated from the coordinates using Microsoft Excel. The tail markers shown in the movies were not used in this study. (DOCX) [file pone.0282277.s003.docx]

S1 Information. Creation of an analytical model and extraction of the head and body coordinates of swimming medaka from movies via DeepLabCut. DeepLabCut (version 2.0) was used to create an analytical model of medaka swimming and to extract the coordinates of the head and body positions. First, 19 frames were automatically extracted using the “kmeans” algorithm from the video of one wildtype medaka. In these frames, the head position was labeled between the left and right eyes. The body position was labeled between the left and right pectoral fins. The size of each marker was 5 µm. Nineteen frames were used as the training dataset in DeepLabCut. Next, DeepLabCut was trained 50,000 times to recognize the position of the head and body of the medaka in the movie based on the training dataset, and an analysis model of medaka swimming was generated. The analytical model of medaka swimming was used to analyze all movies of wildtype and *rad50^Δ2/+^* medaka, and the head and body coordinates were extracted. The root mean square values were calculated from the coordinates using Microsoft Excel. The tail markers shown in the movies were not used in this study.
